# African Genetic Representation in the Context of SARS-CoV-2 Infection and COVID-19 Severity

**DOI:** 10.3389/fgene.2022.909117

**Published:** 2022-05-10

**Authors:** Desiree C. Petersen, Chrystal Steyl, Denise Scholtz, Bienyameen Baker, Ibtisam Abdullah, Caitlin Uren, Marlo Möller, and for The COVID-19 Host Genetics Project

**Affiliations:** ^1^ DSI-NRF Centre of Excellence for Biomedical Tuberculosis Research, South African Medical Research Council Centre for Tuberculosis Research, Division of Molecular Biology and Human Genetics, Faculty of Medicine and Health Sciences, Stellenbosch University, Cape Town, South Africa; ^2^ Division of Haematological Pathology, Department of Pathology, Faculty of Medicine and Health Sciences, Stellenbosch University and NHLS Tygerberg Hospital, Cape Town, South Africa; ^3^ Centre for Bioinformatics and Computational Biology, Stellenbosch University, Stellenbosch, South Africa

**Keywords:** African genomics, genetic susceptibility, SARS-CoV-2 infection variability, COVID-19 severity, COVID-19 genetic associations, limited African data

## Introduction

**FIGURE 1:**
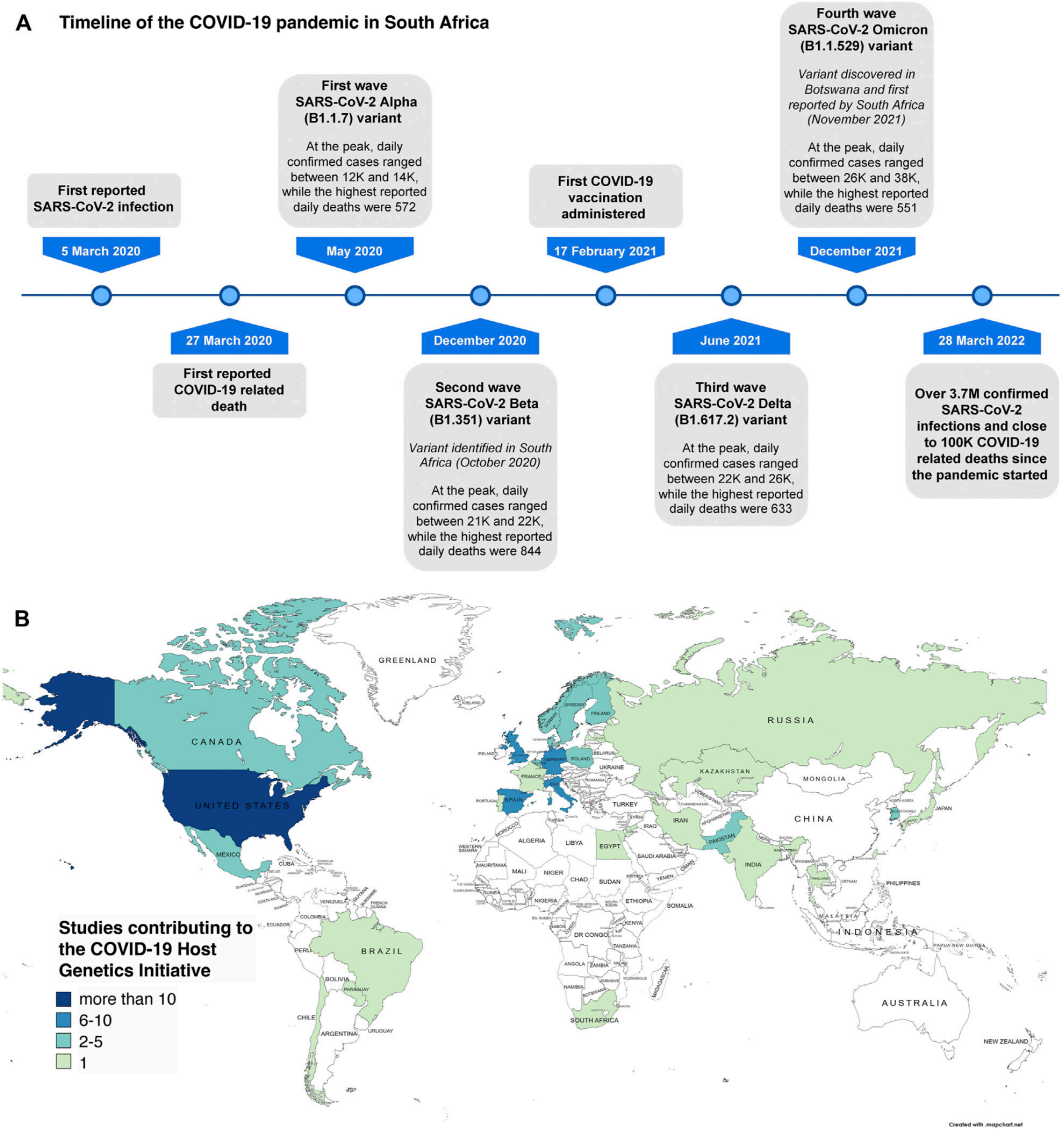
SARS-CoV-2 variant timepoints in South Africa and limited human genetic studies in Africa. **(A)** indicates the significant timepoints where SARS-CoV-2 variants emerged and how this shaped the direction of the COVID-19 pandemic in South Africa (created using data from https://covid19.who.int/region/afro/country/za). **(B)** shows the partners of the COVID-19 Host Genetics Initiative (adapted from https://www.covid19hg.org/partners/). Our own research project entitled, “Host genetic factors contributing to susceptibility to COVID-19 in South Africans” is a registered study with this international effort.

Towards the end of 2019, the world faced the emergence of the Coronavirus Disease 2019 (COVID-19) pandemic caused by the severe acute respiratory syndrome coronavirus 2 (SARS-CoV-2). Up to 28 March 2022, SARS-CoV-2 resulted in over 480 million infections and has been the cause of death in approximately 6.1 million individuals (World Health Organization, 2022a). South Africa has not remained unscathed by the pandemic, having more than 3.7 million COVID-19 cases, and nearing close to 100,000 COVID-19 related deaths (World Health Organization, 2022b) with the introduction of different SARS-CoV-2 variants at various timepoints (Figure 1A). The risks of overwhelmed healthcare systems and an increasing mortality rate have urged for a large amount of research devoted to this disease since much remains unknown (Else, 2020). One of the knowledge gaps is the significant inter-individual variability of host responses demonstrated among SARS-CoV-2 infected individuals. This variability ranges from asymptomatic carriers to individuals who develop severe and, in some cases, lethal COVID-19. Although it has been shown that individuals older than 55 years and those with underlying comorbidities are at higher risk of severe disease, it does not explain the full extent of the variability (Meyts et al., 2020; Zhang et al., 2020; Zhou et al., 2020). A small percentage of younger and relatively healthy individuals also appear unable to control SARS-CoV-2 infection and require medical intervention (van der Made et al., 2020; Grolmusz et al., 2021). Therefore, in addition to considering the pathophysiology, transmissibility and disease severity caused by different SARS-CoV-2 variants, host genetic factors have been proposed as a possible explanation for this residual inter-individual variability (Meyts et al., 2020; Zhang et al., 2020; Guilger-Casagrande et al., 2021; Triggle et al., 2021; Velavan et al., 2021). Human genetic studies to date, mainly performed in Eurasian populations, have identified genetic variants associated with severe COVID-19. As is the case with most disease-associated human genetic studies, many first world countries have been at the forefront of publishing on the COVID-19 topic (Ellinghaus et al., 2020; van der Made et al., 2020; Zhang et al., 2020; Pairo-Castineira et al., 2021; Velavan et al., 2021). This is likely attributed to the availability of large existing biobanks making rapid COVID-19 human genetic research possible (COVID-19 Host Genetics Initiative, 2020; Zhang et al., 2020; Pairo-Castineira et al., 2021; Kousathanas et al., 2022). South Africa and the rest of the African continent has, however, not been able to contribute human genetic data at the same pace resulting in limited information being available for local African populations. Furthermore, African populations show the greatest genetic diversity and extrapolating the results obtained from Eurasian population studies might prove to be irrelevant or may result in the exclusion of significant genetic variants when establishing COVID-19 genetic risk profiles for these understudied populations (Martin et al., 2018).

## Human Genetic Studies of SARS-CoV-2 Infection and COVID-19 Severity

Publications focusing on the role of host genetic factors in determining susceptibility to SARS-CoV-2 infection and COVID-19 severity have included epigenetic, mitochondrial, candidate gene, and genome-wide association studies (GWAS) as well as the use of whole exome sequencing and whole genome sequencing (WGS) (Ellinghaus et al., 2020; Zhang et al., 2020; Chlamydas et al., 2021; Kgatle et al., 2021; Scozzi et al., 2021; Sen et al., 2021; Velavan et al., 2021; Wu et al., 2021). One of the earlier human genetic studies included a GWAS of SARS-CoV-2 respiratory failure, which identified associations with the ABO blood locus and a chromosome 3 gene cluster (*SLC6A20*, *LZTFL1*, *CCR9*, *FYCO1*, *CXCR6* and *XCR1*) in Italian and Spanish populations (Ellinghaus et al., 2020). Another study of 659 hospitalized COVID-19 patients identified rare and likely pathogenic genetic variants at 13 loci, known to influence immunity to the influenza virus, associated with life-threatening COVID-19 pneumonia (Zhang et al., 2020). Several studies have since confirmed these genetic associations and have identified additional variants in *MUC5B, OAS3, OAS1, TLR7* and *TYK2*, which are associated with critical illness and severity (Pairo-Castineira et al., 2021; Velavan et al., 2021). The largest study to date consists of 49,562 COVID-19 cases representing 19 countries. This includes findings from three GWAS meta-analyses, performed by the COVID-19 Host Genetics Initiative (HGI), showing 13 significant genetic loci to be associated with either susceptibility to SARS-CoV-2 infection or severe outcomes of COVID-19 (COVID-19 Host Genetics Initiative, 2021). A large United Kingdom case-control cohort used WGS and identified novel variants in 16 genes that are associated with critical COVID-19 (Kousathanas et al., 2022). Many of the genes associated with COVID-19 in the above-mentioned studies, are implicated in fundamental pathophysiological processes, with the majority affecting immune response pathways (Ellinghaus et al., 2020; Zhang et al., 2020; COVID-19 Host Genetics Initiative, 2021; Pairo-Castineira et al., 2021; Velavan et al., 2021; Kousathanas et al., 2022).

Previous host genetic research has indicated that SARS-CoV-2 susceptibility and COVID-19 severity seem to be polygenic. It has therefore been proposed that calculating polygenic risk scores (PRS) could be useful as it allows for the detection of individuals at high risk. (Grolmusz et al., 2021; Velavan et al., 2021). A study by Prakrithi *et al.* calculated the PRS of previously identified COVID-19 associated single nucleotide polymorphisms (SNPs) in different Indian sub-populations, which allowed them to identify populations at higher risk of COVID-19-related deaths, and thereby provide support for vaccination prioritization in those specific populations (Prakrithi et al., 2021). In addition, a group from Australia designed a model to predict an individual’s COVID-19 severity risk, showing that including genetic and clinical risk factors, as opposed to only using age and sex, increases the accuracy for risk discrimination by 111%. (Dite et al., 2021). Predictive scores and models do have some limitations, including being population specific as the allele frequencies and linkage disequilibrium (LD) patterns used in these calculations could differ significantly across and within populations (Sirugo et al., 2019; Grolmusz et al., 2021; Prakrithi et al., 2021).

## Lack of COVID-19 Genetic Studies in Africa–Why This is an Area of Concern

In contrast to the rest of the world, people living in Sub-Saharan Africa appear to be less prone to develop severe COVID-19 (Adams et al., 2021). This was surprising as the risk of developing severe COVID-19 was predicted to be elevated in Africa due to the high incidence of other infectious diseases such as HIV/AIDS and tuberculosis (TB), as well as the increased prevalence of non-communicable diseases such as hypertension and type 2 diabetes mellitus in certain African countries, including South Africa (Dave et al., 2021; Jassat et al., 2021). Several main hypotheses, including Sub-Saharan Africa’s demographic distribution relating to age and sex; the lack of SARS-CoV-2 testing; the shortage of long-term care facilities that pose a higher risk for transmitting infectious and communicable diseases; existing protection due to previous exposure to locally circulating coronaviruses; and effective public health response supported by African governments, may have resulted in reduced morbidity and mortality rates (Adams et al., 2021). It is also possible that certain diseases or other prior infections may have an unexpected protective effect against severe COVID-19, as has been shown in the case of malaria (Altable and de la Serna, 2021; Osei et al., 2022), however, variable COVID-19 severity could also be explained by genetic differences present in these African populations (Adams et al., 2021).

Populations in Africa are highly diverse and represent some of the oldest extant populations e.g., the Khoe-San (Schuster et al., 2010; Pickrell et al., 2012; Petersen et al., 2013; Uren et al., 2016; Uren et al., 2017). In addition, modern migration routes have allowed for admixture between previously geographically distinct populations and have led to highly genetically heterogeneous populations where in some cases, there are five contributing ancestral populations (Campbell and Tishkoff, 2010; de Wit et al., 2010; Patterson et al., 2010; Petersen et al., 2013; Uren et al., 2016; Uren et al., 2017). Furthermore, African genomes have novel characteristics i.e., a larger number of novel variants and shorter more heterogeneous LD (Sirugo et al., 2019; Vergara-Lope et al., 2019). In addition, best practices as implemented by standard data analysis pipelines lack efficiency and accuracy in African populations (Uren et al., 2020). This diversity and unique genomic characteristics have phenotypic implications resulting from unique genetic factors influencing both simple and complex phenotypes such as altered disease susceptibility (Campbell and Tishkoff, 2010; Patterson et al., 2010; Uren et al., 2017). To date, however, the majority of human genetic data generated, particularly those investigating genotype-phenotype correlations, has been biased towards Eurasian populations, as has also been noted for COVID-19 research (Sirugo et al., 2019; Ellinghaus et al., 2020; van der Made et al., 2020; Zhang et al., 2020; Velavan et al., 2021; Pairo-Castineira et al., 2021). International consortiums, including the COVID-19 HGI, the COVID-19 Human Genetics effort (HGE), and the Genetics Of Mortality In Critical Care (GenOMICC) have promoted the sharing of data to facilitate the inclusion of large study cohorts from multiple populations for ongoing meta-analyses (COVID-19 Host Genetics Initiative, 2020; COVID Human Genetic Effort, 2022; GenOMICC, 2022). Although the existing international consortiums and several research groups aim to bridge this gap, much more is needed. Only one of the 119 partner studies that contribute to the COVID-19 HGI (Figure 1B) (COVID-19 Host Genetics Initiative, 2022) and two of the 276 centers that contribute to the COVID HGE (COVID Human Genetic Effort, 2022) currently include populations from Sub-Saharan Africa. By considering the unique aspects of African genomes, and preliminary findings suggesting novel COVID-19 susceptibility markers in African populations, a larger emphasis needs to be placed on generating and analyzing genetic data that is representative of Africa. There is still a lack of suitable genomic references and statistical tools for interpretation of African genetic data since most of the existing references and other related tools are based on Eurasian populations (Martin et al., 2018; Sirugo et al., 2019). Results from African-based COVID-19 host genetic studies will not only benefit the populations in which they occur, but rather all populations with African ancestral contributions.

## Discussion

Although viral genome sequencing and the rapid discovery of new viral variants were exceptionally successful in South Africa (Tegally et al., 2021; Wilkinson et al., 2021; Viana et al., 2022), the same is not completely true for the host genome sequencing, even though the expertise and infrastructure for human WGS is available (Glanzmann et al., 2021). The relatively cheaper viral sequencing compared to human WGS, and the complexity of human genome data compared to the viral genome data are partly the reason, together with unique ethical considerations for human genetic research in Africa, difficulty in obtaining written informed consent, and the challenges faced with sample collection (Martin et al., 2018; Marshall et al., 2022). Unfortunately, genetic research on the African continent continues to be hindered by limited resources, including delays to obtain ethical approval for human genetic studies and inadequate infrastructure (Martin et al., 2018; Hamdi et al., 2021). Despite the substantial investment made by both local and international funding organizations for human genetic studies in the more recent years (H3Africa Working Group, 2011; Choudhury et al., 2020; Maxmen, 2020), this alone is insufficient to generate large-scale genotyping and sequencing data in Africa. This is partially due to higher technology and reagent costs compared to most first world countries as well as additional expenses for bioinformatics processing and data storage hardware (Hamdi et al., 2021; Mboowa et al., 2021).

In addition to forming international collaborations, Africa needs to establish large biobanks, including the collection of phenotype information and the option to recontact participants for additional informed consent, in the case of broad consent not being favored in certain countries (Moodley and Singh, 2016; Tindana et al., 2019). This will ensure that the continent also swiftly contributes to human genetic studies in the case of future pandemics. Genetic findings for African populations may provide significant insights into the disease pathogenesis, which could lead to developing suitable therapeutic interventions that could assist with the management of COVID-19 in many resource-poor countries. This includes prioritized vaccination of genetically at-risk individuals to avoid unfavorable COVID-19 outcomes. At this stage, due to the many above-mentioned shortcomings, Africa continues to remain behind in matching the host genetic research efforts made by international collaborators on a global scale.

## COVID-19 Host Genetics Project

Marlo Möller, Desiree C. Petersen, Craig J. Kinnear, Caitlin Uren, Brigitte Glanzmann, Elouise Kroon, Richard Glashoff, Shane Murray, Judith Hornby Cuff, Hendrik La Grange, Natrisha Damons, Helena Cornelissen, Zivanai Cuthbert Chapanduka, Ibtisam Abdullah, Deepthi Raju Abraham, Helena Rabie, Chrystal Steyl, Denise Scholtz, Annecke Vermeulen, Tongai Maponga, Kate Webb, Sian Hemmings, Gert van Zyl, Aubrey Shoko, Ansie Wichers and Sihaam Boolay from Stellenbosch University (Divisions of Molecular Biology and Human Genetics, Haematological Pathology, Medical Microbiology and Immunology, Rheumatology, and Medical Virology, Departments of Paediatrics and Child Health and Psychiatry), the University of Cape Town (Department of Paediatrics and Child Health), the Centre for Proteomic and Genomic Research (CPGR), Artisan Biomed, the South African Medical Research Council Centre for Tuberculosis Research, the South African Medical Research Council Genomics Centre and the National Health Laboratory Service (NHLS).
